# Subdiffraction-resolution fluorescence imaging of immunological synapse formation between NK cells and *A. fumigatus* by expansion microscopy

**DOI:** 10.1038/s42003-021-02669-y

**Published:** 2021-10-04

**Authors:** Nora Trinks, Sebastian Reinhard, Matthias Drobny, Linda Heilig, Jürgen Löffler, Markus Sauer, Ulrich Terpitz

**Affiliations:** 1 Department of Biotechnology and Biophysics, Theodor-Boveri-Institute, Biocenter, Julius Maximilian University, Würzburg, Germany; 2 Department of Internal Medicine II, WÜ4i, University Hospital Würzburg, Würzburg, Germany

**Keywords:** Fluorescence imaging, Super-resolution microscopy, Biological fluorescence, Imaging the immune system, Infectious diseases

## Abstract

Expansion microscopy (ExM) enables super-resolution fluorescence imaging on standard microscopes by physical expansion of the sample. However, the investigation of interactions between different organisms such as mammalian and fungal cells by ExM remains challenging because different cell types require different expansion protocols to ensure identical, ideally isotropic expansion of both partners. Here, we introduce an ExM method that enables super-resolved visualization of the interaction between NK cells and *Aspergillus fumigatus* hyphae. 4-fold expansion in combination with confocal fluorescence imaging allows us to resolve details of cytoskeleton rearrangement as well as NK cells’ lytic granules triggered by contact with an RFP-expressing *A. fumigatus* strain. In particular, subdiffraction-resolution images show polarized degranulation upon contact formation and the presence of LAMP1 surrounding perforin at the NK cell-surface post degranulation. Our data demonstrate that optimized ExM protocols enable the investigation of immunological synapse formation between two different species with so far unmatched spatial resolution.

## Abstract (web summary)

Trinks et al developed a protocol that enables super-resolved visualisation of the interaction between natural killer cells and Aspergillus fumigatus hyphae by expansion microscopy. They describe in detail steps of the formation of the immunological synapse including cytoskeleton re-arrangement, granule convergence and translocation of LAMP1, demonstrating the utility of the method.

## Introduction

Spores of *Aspergillus* species are ubiquitously distributed in the air and humans inhale thousands of fungal spores per day^1^. In healthy individuals, cilia and mucus will remove the majority of entering spores from the lung. Only a small fraction of spores enter the alveoli, where innate immune cells build up the first line of defense against invading fungi^2,3^.

Nevertheless, immunocompromised patients, e.g., receiving hematopoietic stem cell transplantation (HSCT) or cancer therapy^4–6^, patients with genetic defects^7^, or patients suffering from grave diseases like influenza or Covid19^8^, may develop severe fungal infections such as invasive aspergillosis (IA) that often heavily infects the lung. Even if antifungal agents and strategies of antifungal prophylaxis are supplied, there are mortality rates in the range of 30–35% or even higher^9^.

Natural killer (NK) cells that contribute to 5–20% of the lymphocytes in the blood were recently shown—based on mouse models as well as in clinical studies—to play an important role in the clearance of fungal infections^10–12^. Patients receiving HSCT are at higher risk of developing IA due to reduced NK-cell counts and delayed NK-cell reconstitution^13^. Additionally, impaired migration of pulmonary NK cells in neutropenic mice favored the development of IA^14^.

NK cells appear in two major subsets, either CD56^bright^CD16^low^ that produce high amounts of cytokines or CD56^dim^CD16^+^ that efficiently lyse target cells. NK cells form immunological synapses (IS) with their target cells, playing a pivotal role in the killing mechanism. The IS has been analyzed in detail for the interaction of NK and cancer cells^15^, but little is known for the IS formed between NK cells and *Aspergillus fumigatus* hyphae^11^. IS formation strongly depends on the polymerization of actin^16^ and is impaired in NK cells obtained from allogeneic HSCT recipients, recovering within 180 days post-HSCT^17^.

Similar mechanisms are used for the defense against cancer cells and fungi^12,18^, whereby polarized degranulation is the central mechanism in the killing activity of NK cells^15^. Cytotoxic compounds, especially granulysin (also known as NK-lysin), perforin, and granzymes are actively transported via granules toward the IS and released in the synaptic cleft. The 9 kDa form of granulysin is enriched in cytotoxic granules of NK cells, transported towards the IS^19^, and released by receptor-mediated granule exocytosis to affect target cells. High granulysin concentrations of >1 μM are required for killing fungi in vitro^20^. While granulysin induces pore-formation in membranes lacking cholesterol (bacteria, fungi, and lipid rafts in mammalian cell membranes), perforin targets cholesterol-enriched membranes like cancer cells or immune cells infected with intracellular parasites. Pore-formation enables granzymes, a family of proapoptotic proteases, to enter into target cells. In consequence, apoptosis of target cells is induced^21^. Though granulysin shows high activity against microbes, NK-cell killing of *Cryptococcus* is mediated by perforin^22^.

For a better understanding of the processes underlying IS formation and polarized degranulation it is beneficial to visualize those proteins by fluorescence imaging. In the recent past, fluorescence microscopy experienced a number of improvements and inventions increasing the resolution far below the diffraction limit of light^23,24^.

Most super-resolution microscopy techniques gain their increased resolving power from the improvement of the optical components or they exploit distinct photophysical properties of the sample to reconstruct super-resolved images after computational analysis. A completely different concept underlies expansion microscopy (ExM)^25^. ExM increases resolution and reveals subdiffraction information without requiring the use of high-sophisticated microscopes. Instead, the sample itself is physically expanded by means of polyacrylamide hydrogels that enable, under ideal conditions, isotropic expansion of the sample.

Briefly, cells are fixed and fluorescently stained following well-established standard protocols. Using methacrylic acid *N*-hydroxysuccinimide ester (MA-NHS), acryloyl-X (AcX), or glutaraldehyde (GA)^26^ amino groups are modified to enable incorporation of the cellular components into a polyacrylamide hydrogel. After enzymatic treatment with proteinase K^27^, a step known as homogenization, the gel is expanded in pure water. Consequently, also the distances between fluorophores are uniformly extended, leading to an effective lateral resolution of ∼60 nm by confocal microscopy after fourfold expansion^25^ and above with direct 10x or iterative 20x expansion^28–30^. Further increase in resolution can be gained when ExM is combined with other super-resolution microscopy methods^31^.

ExM has been used successfully for super-resolution imaging of mammalian cells^32–34^ virus particles^35^, bacterial pathogens^36^, and plants^37^. Recently, ExM has been optimized for cell biology studies in fungi including *Ustilago maydis* sporidia and hyphae of *Fusarium oxysporum* or *A. fumigatus*, and used to investigate subcellular structures with an estimated spatial resolution of ~30 nm^38^. However, ExM has never, to the best of our knowledge, been used to investigate the interaction of immune cells with fungi.

In the present study, we developed a protocol that enables the simultaneous expansion and imaging of fungi and their interactions with immune cells with so far unmatched spatial resolution. We focused our investigations on the visualization of processes involved in immunological synapse formation between NK cells and *A. fumigatus* hyphae.

## Results

### A protocol for simultaneous expansion of NK cells and fungi

**Fig. 1:**
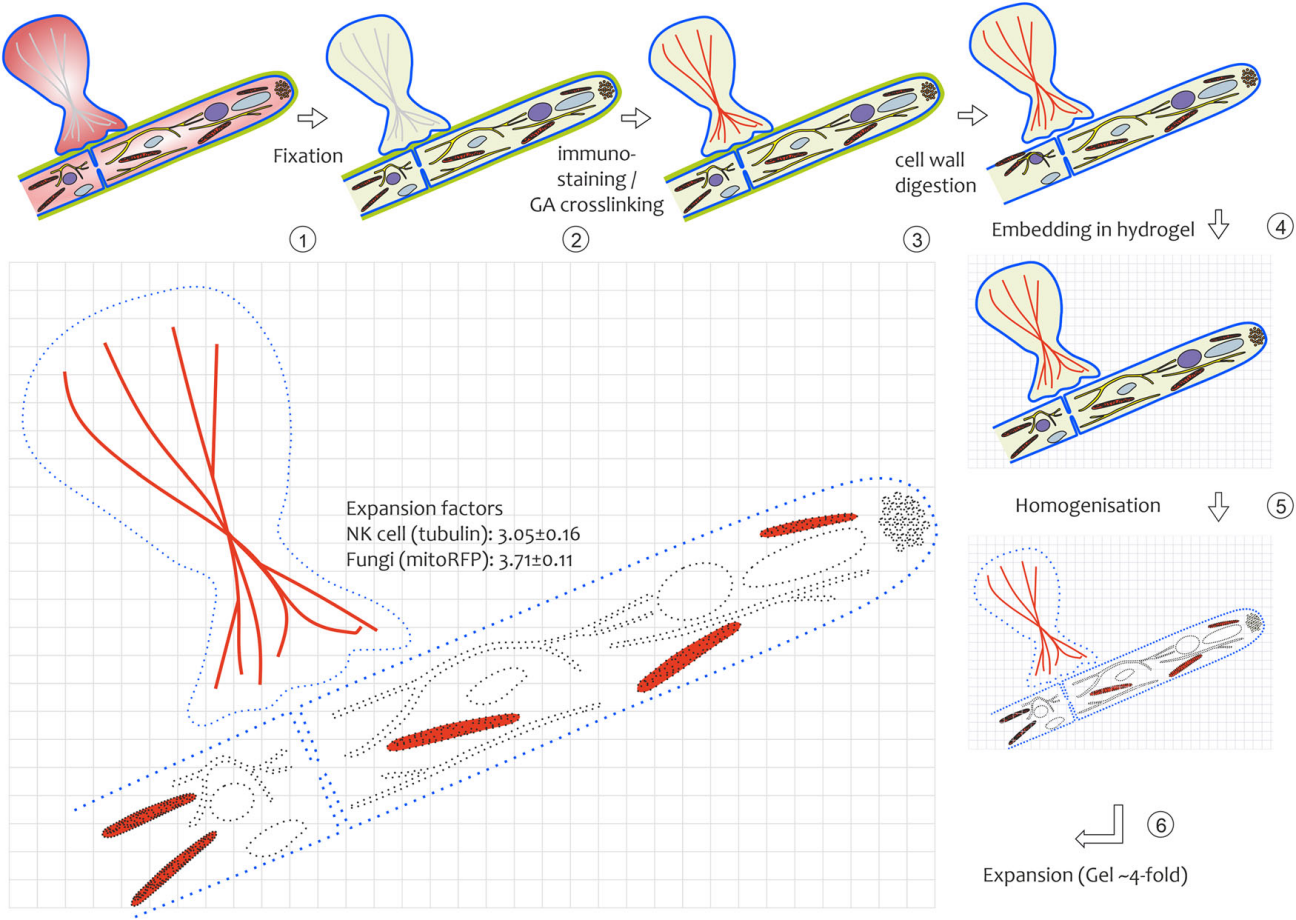
Schematic workflow for NK cell/*A. fumigatus* coculture preparation for ExM. (1) After coculture the sample was fixed specifically, regarding the structure of interest. (2) Immune fluorescence staining and signal amplification via prim/sec ab staining. For visualization of *A. fumigatus* hyphae, a red-fluorescent-protein-expressing strain was used (RFP-tagged mitochondria). NK cells were immunolabeled with primary and secondary antibodies for different target structures of interest. Anchoring of fluorescent labels was performed by postfixation with GA. (3) Removal of fungal cell wall components, using enzymatic digestion. (4) Sample embedding in a hydrogel, called gelation. (5) Cutting of peptide bonds, using proteinase K digestion. A process called homogenization, prior to gel swelling. (6) Expansion in water. Gel swelling due to the absorbance of water molecules (expansion factor ~4). The resulting expansion factors for tissues were deferred between fungi (3.71 ± 0.11, mean + SD) and NK cells (3.05 ± 0.16).

ExM of miscellaneous organisms within the same sample remains challenging. Due to differences in organisms’ morphology and texture, each species requires distinct treatments during the ExM procedure. Dealing with the fact, that the interaction of immune cells with fungi urgently requires detailed investigations with high spatial resolution, we evaluated the possibility of combining all steps that are required for ExM of either fungi or mammalian cells in one mutual procedure using the following workflow (Fig. 1).

In the first step, NK cells were cocultured with an *A. fumigatus* strain constitutively expressing a fluorescent protein located in mitochondria at an MOI of about 0.5. Coculture time depended on the target structure of interest, with 3–3.5 h for cytoskeleton structures and 5.5 h for degranulation events and was chosen ensuring a pronounced IS formation, but still young hyphae that are more susceptible to cell wall lytic enzymes. The subsequent fixation procedure of cocultures was adapted and optimized for the respective target structures, i.e., cytoskeleton was fixed with triton and GA according to Small et al.,^39^ while granulae were fixed with 0.7% FA and permeabilized with 0.1% saponin, followed by GA fixation after immunostaining. NK cells were labeled by immunostaining or other suitable organelle-specific fluorescent probes. The ratio of primary and secondary antibodies and/or dyes linked to reactive groups was optimized gaining convenient signal amplification. Together with the immunostaining procedure, modification of amino acids providing the linkers for hydrogel-embedding by GA was accomplished^26^.

Expansion of fungi necessitates an additional step that ensures the complete digestion of the complex-organized multicomponent cell wall. It is pivotal to perform fixation before digestion in order to preserve the original shape of the fungal hyphae^38^. After sample embedding overnight (gelation), homogenization was performed using proteinase K. By contrast to fungi that are stabilized by the extracellular cell wall, in mammalian cells the cytoskeleton determines cell shape^40,41^. The dense organization of cytoskeleton structures may require longer treatment with proteinase K than in fungi to ensure isotropic expansion. We obtained satisfying results applying proteinase K digestion for 6 h at room temperature.

Following this protocol, we observed an efficient simultaneous expansion of both, fungal hyphae and NK cells. To verify the expansion factor and to investigate potential structural distortions, we imaged the same NK cells and fungi before and after fourfold expansion. Upon demineralization, the gel expanded by a factor of 3.9 ± 0.13 (Supplementary Fig. 1). Visualizing the same structures before and after expansion, we noticed that expansion occurs not completely isotropic in both species. We used Elastix (SimpleElastix)^42,43^ to compute a similarity transform, that maps the pre-expansion image to the post expansion image using microtubules (NK cells) and mito RFP (fungi) as target structures (see the workflow in Supplementary Fig. 2). For fungi, we determined an average expansion factor of $3.71\pm 0.11$ (SD, *n* = 4; Supplementary Fig. 3). In contrast, for NK cells, the average expansion factor was smaller, $3.05\pm 0.16$ (SD, *n* = 8; Supplementary Fig. 4). This finding is in accordance with Büttner et al., who observed that different cellular compartments exhibit slightly different expansion factors^44^.

Taking Pearson correlation and distortion maps into account, NK cells expanded less but more isotropic. This might be a result of shortening the time for proteinase K treatment in the mutual protocol to preserve the mRFP signal of the co-expanded fungus. Improvement of the homogenization protocol might equalize mechanical properties across the sample and allow for more similar expansion factors.

### Rearrangement of cytoskeleton components during NK-fungus interaction

It is known that the NK-cell cytoskeleton undergoes a significant rearrangement upon interaction with target cells^45,46^. Microtubules play a key role in the directional transport of lytic granules and thus in the cytotoxicity of immune cells^47^. Once the NK cell faces the target cell via PRRs, lytic granules are first actively transported towards the microtubule organization center (MTOC) in a dynein-dependent manner^48^. Thereafter, the MTOC converges towards the immunological synapse. However, the rearrangement of microtubules upon fungal contact has not yet been investigated in detail for the interaction of NK cells and *A. fumigatus* due to the limited spatial resolution of standard fluorescence microscopy.

**Fig. 2:**
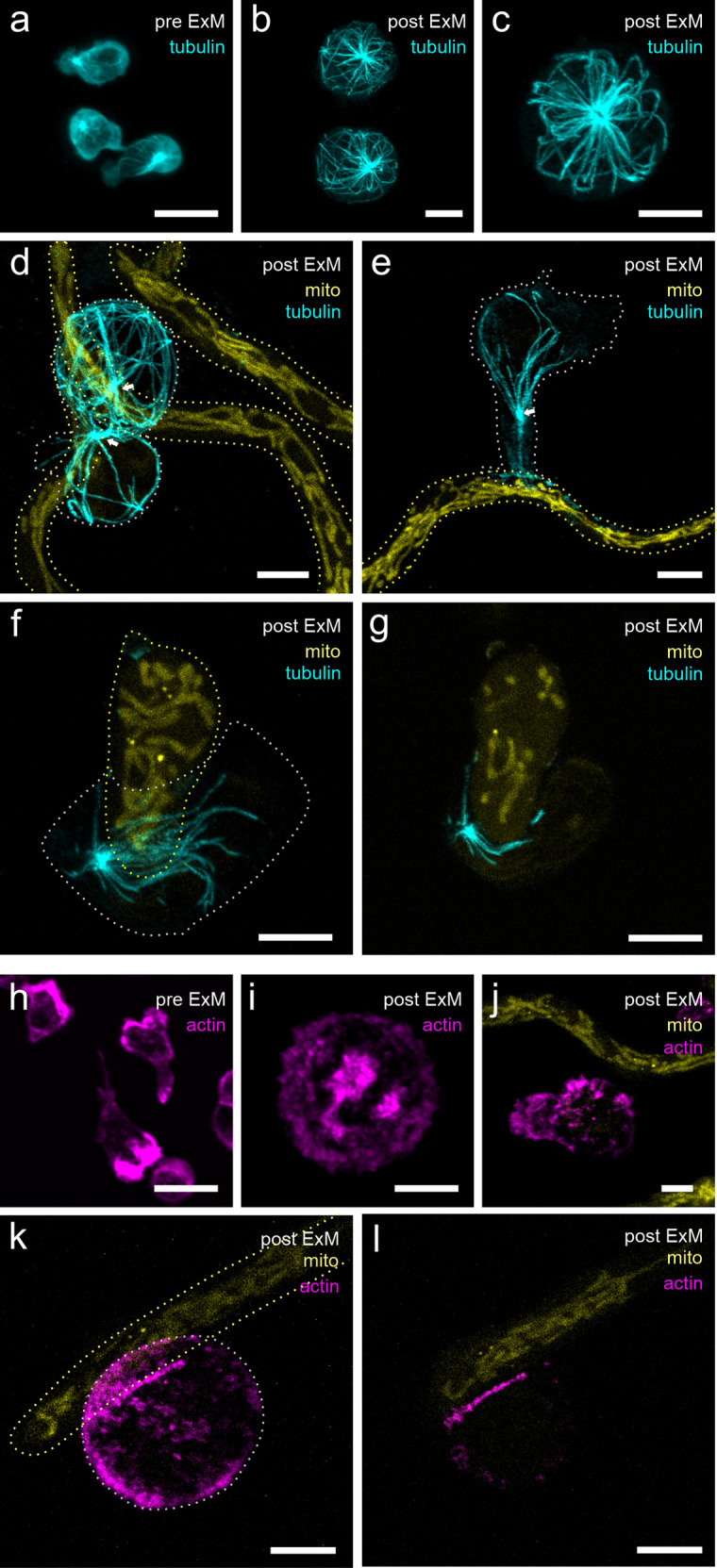
NK cell cytoskeleton visualization by expanding α–tubulin and actin in NK cells alone and co–cultured with *A. fumigatus*. Conventional CLSM images of NK cells settled on PDL–treated coverslips prior to expansion (**a** α–tubulin, **h** actin) and respective ExM images (**b**, **c** α–tubulin, **i** actin) are shown. Note the difference in NK cell size post ~4x expansion. ExM images of NK/*A. fumigatus* co–cultures are shown in **d**–**g** (alpha–tubulin) and **j**–**l** (actin) post 3 h (**e**), 3.5 h (**d**) and 5.5 h (**f**, **g**, **j**–**l**) of co–incubation. NK–cell cytoskeleton structures show a prominent re–organization towards *A. fumigatus* hyphae. Interacting NK cells show MTOC polarization towards *A. fumigatus* hyphae and accumulation of actin at the site of interaction. In contrast, cells that are not yet interacting with the fungus showed equal distribution of the actin signal on the cell surface. All images represent maximal intensity z–projections of the whole image stack, with the exception of panels **g** and **l**, that represent maximal intensity z–projections of only 3 (**g** slices 19–21) or 4 (**l** slices 19–22) slices of the samples shown in panels **f** and **k**, where interaction of the NK cell with the fungus was most pronounced. Dotted lines indicate borders of NK cells (grey) and fungal hyphae (yellow). Representative images out of 5 (**a**–**h**) and 2 (**i**–**l**) biological replicates. Scale bars, 10 µm (**a**–**l**).

Therefore, we tested the suitability of our ExM protocol for visualization of the cytoskeleton in NK cells interacting with *A. fumigatus*. In general, within the same sample, the state of IS development differed between NK cell/hyphae conjugates. In addition, we noticed variations in cocultures of different NK-cell donors, where NK cells seemed to be more or less active against *A. fumigatus*. The fixation of microtubules was optimal in the presence of triton and high GA-content^39^. The naive NK cells showed an astral microtubule organization with the highest density at the MTOC (Fig. 2a–c). The intact microtubule network became visible in much more detail in the expanded cell (Fig. 2b, c). ExM enabled us to show MTOC migration toward the interaction site of fungus and NK cells (Fig. 2d–g), similar as described for cancer cells or other fungal species like *Cryptococcus neoformans*^49^.

Recently, we noticed redistribution of actin towards the interaction site between fungus and NK cell^16,17^. Therefore, we next compared the actin distribution in naive NK cells (Fig. 2h, i) and NK cells interacting with the fungus after 5.5 h cocultivation (Fig. 2j–l). In contrast to microtubules, the visualization of actin by ExM remained, however, challenging. Native phalloidin lacks reactive groups necessary for anchoring into the hydrogel, making it useless for super-resolution imaging of actin filaments by ExM^50^. However, we tested different alternative protocols and succeeded in using a phalloidin derivate that was biotinylated via a linker (phall-XX-biotin). Finally, the combination with fluorescently labeled streptavidin (ATTO 643-streptavidin) enabled us to visualize actin enrichment in NK cells targeting *A. fumigatus* hyphae by ExM (Fig. 2k, l).

Similar, as with tubulin also the actin distribution beneath the NK-cell plasma membrane became visible in the expanded sample in more detail (Fig. 2i) that was not distinguishable in conventional, unexpanded samples (Fig. 2h). Nevertheless, the visualization of actin in the direct environment of the membrane still needs further improvement and could benefit from future phalloidin-variants such as trifunctional linkers and other alternative approaches optimized for ExM^50,51^. Indeed, we observed an accumulation of actin in the IS region of those NK cells that interacted with *A. fumigatus*. Single filopodia at naive NK cells were hardly distinguished whereas advantageously, autofluorescence of the expanded samples was reduced.

Of note, in regions of accumulated actin, the morphology of fungal mitochondria appeared somehow changed (Fig. 2k) or mitochondria were even absent, potentially indicating that fungal health was impaired by the NK cell in the interaction region^52,53^.

### Polarized degranulation

Perforin and granulysin play a pivotal role in the killing activity of NK cells^54^. They are enriched in secretory lytic granules that fuse with the presynaptic membrane, releasing these cytotoxic proteins into the synaptic cleft. Both proteins show cytolytic activity by forming pores in the membranes of the target cells after oligomerization.

**Fig. 3:**
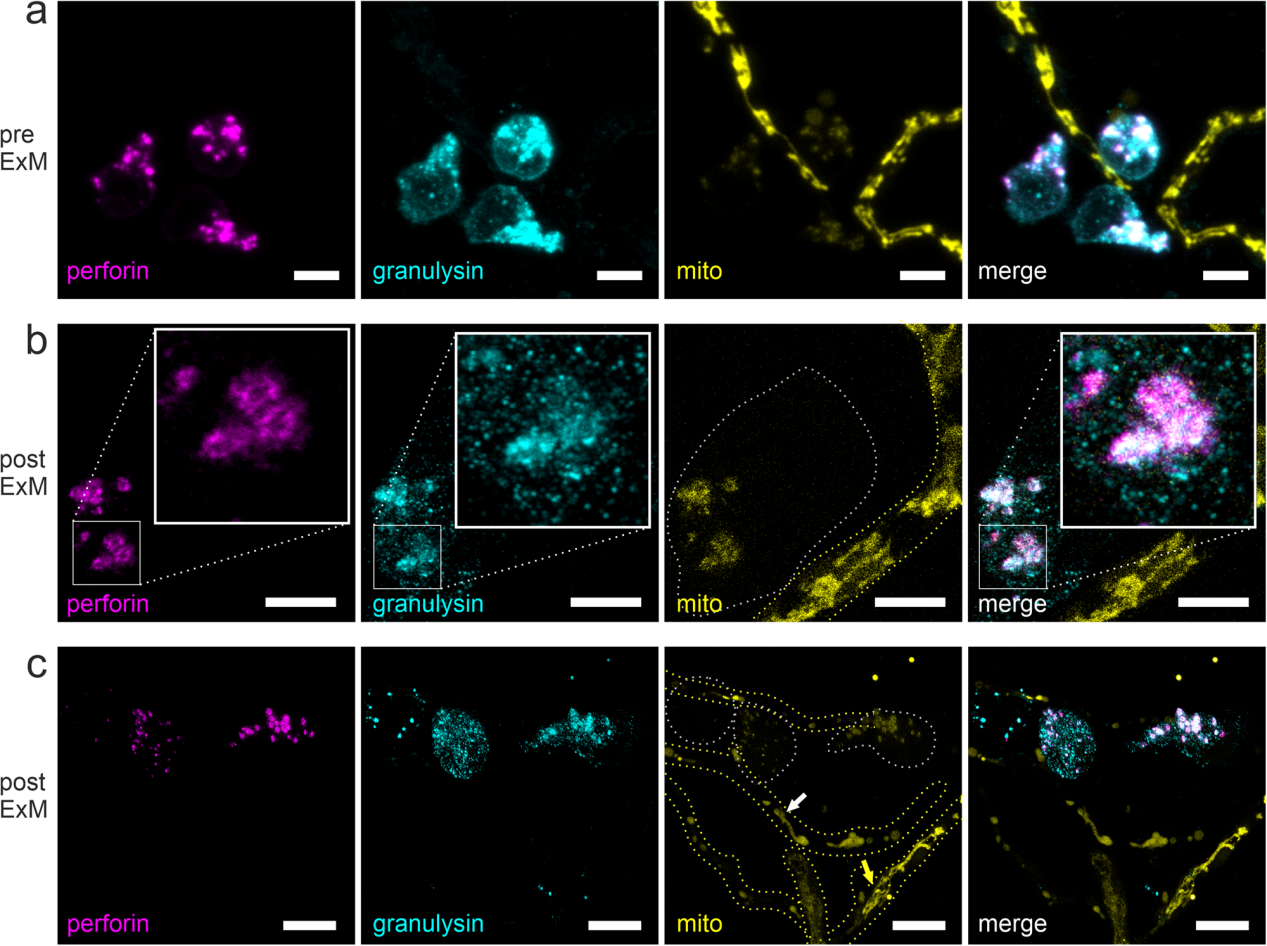
Visualization of NK-cells lytic granules after 5.5 h coculture with *A. fumigatus* hyphae. Perforin (magenta) and granulysin (cyan) were visualized by antibody staining, fungal hyphae via RFP-expressing mitochondria (yellow). **a** Conventional CLSM images prior to expansion. Perforin and granulysin are partially overlapping in round-shaped granules. **b** ExM images. Post ~4x expansion, labeled perforin appears as a ring-like structure, whereas granulysin was also distributed throughout the NK cell. Insets in panel **b** represent a detailed view of the indicated region. **c** Effect of degranulation. In the direct environment of degranulating NK cells, mitochondria appear distorted or even absent (white arrow) in comparison to hyphae more distant (yellow arrow). All images represent maximum intensity z-projections. Dotted lines indicate borders of NK cells (gray) and fungal hyphae (yellow). Representative images of at least three biological replicates. Scale bars, 5 µm (**a**), 10 µm (**b**), and 25 µm (**c**).

Thus, we visualized perforin and granulysin in NK cells by CLSM before and after expansion (Fig. 3). According to the previous theoretical consideration, we observed colocalization of perforin and granulysin in NK cells, with both methods. We analyzed the colocalization of granulysin and perforin by computing Manders, Pearson, and Spearman correlation indices for *n* = 9 samples in pre- and post expansion out of three biological replicates. For pre-expansion, we obtained Spearman (0.851 ± 0.051), Manders (0.844 ± 0.048), and Pearson (0.787 ± 0.070). For post expansion, we obtained Spearman (0.835 ± 0.066), Manders (0.772 ± 0.082), and Pearson (0.71 ± 0.11). Interestingly, perforin appeared to be arranged in a ring-like shape that could hardly be resolved by conventional CLSM (Fig. 3a) but with ExM (Fig. 3b). In addition, granulysin though not that obvious exhibited a ring-like appearance. The granulysin signal was also found throughout the NK cell, most likely caused by the 15-kDa form that is located in different granules and lacks cytotoxic activity^19^.

Furthermore, in samples where perforin and granulysin were detected, the mitochondria in the *Aspergillus* hyphae showed impaired morphology or were even absent (Fig. 3c, see also pre-ExM images in Supplementary Fig. 5). Similar effects on the mitochondria morphology were recently described in *A. fumigatus* hyphae attacked by human granulocytes^53^.

Lysosome-associated membrane protein 1 (LAMP1), also known as CD107a, has been established as a marker for NK-cell degranulation. Nevertheless, the role in NK-cell biology, especially the release of cytotoxic enzymes is yet not well understood. Interestingly, with a lack of LAMP1, granules showed shorter tracks, smaller displacement, and decreased velocity^55^. Polarized degranulation can be visualized in the presence of brefeldin A (BFA), a fungal metabolite that blocks the transport from the endoplasmic reticulum to the Golgi apparatus and by that prevents secretion of proteins^56^. The release of preformed NK-cell granules is not affected while cytokine release and recycling of effector molecules is impaired^57^.

**Fig. 4:**
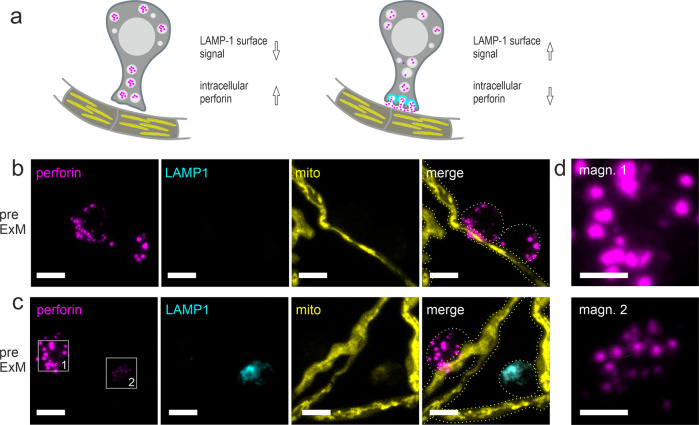
NK-cell degranulation assay. **a** Schematic figure of NK-cell degranulation. Non-degranulated NK cells appear negative for degranulation marker LAMP1 (cyan), but positive for perforin (magenta). In contrast, after NK-cell degranulation in the presence of *A. fumigatus* hyphae and BFA, intracellular perforin is reduced and surface LAMP1 can be detected. **b**–**d** Visualization of surface LAMP1 (cyan) and intracellular perforin (magenta) in presence of the *A. fumigatus* (RFP-expressing mitochondria, yellow) with standard CLSM before (**b**) and after (**c**) degranulation. In **c** a degranulated NK cell (box 2) that appears positive for both, LAMP1 marker protein and perforin is shown together with a cell before degranulation (box 1). **d** Magnification of the perforin signal shown in **c**. Note the difference in sizes and signal intensities for perforin post degranulation. NK cells and *A. fumigatus* hyphae were cocultured for 5 h. All images shown represent maximum intensity z-projections. Dotted lines indicate borders of NK cells (gray) and fungal hyphae (yellow). Representative images of two biological replicates. Scale bars, 5 µm (**b**, **c**) and 2 µm (**d**).

Before degranulation, one would expect the intracellular accumulation of perforin in NK cells and the absence of LAMP1 on the surface. Upon degranulation, the intracellular perforin should decrease and LAMP1 be prominently exposed at the cell surface (Fig. 4a). We analyzed NK cells cocultured with *A. fumigatus* using CLSM and a protocol that avoids intracellular staining of LAMP1 in living, intact NK cells. As expected, we found NK cells with a strong perforin signal but no LAMP1 signal indicating the status before degranulation (Fig. 4b). However, other NK cells exhibited a decrease in perforin signal strength associated with a strong surface localization of LAMP1 (Fig. 4c).

**Fig. 5:**
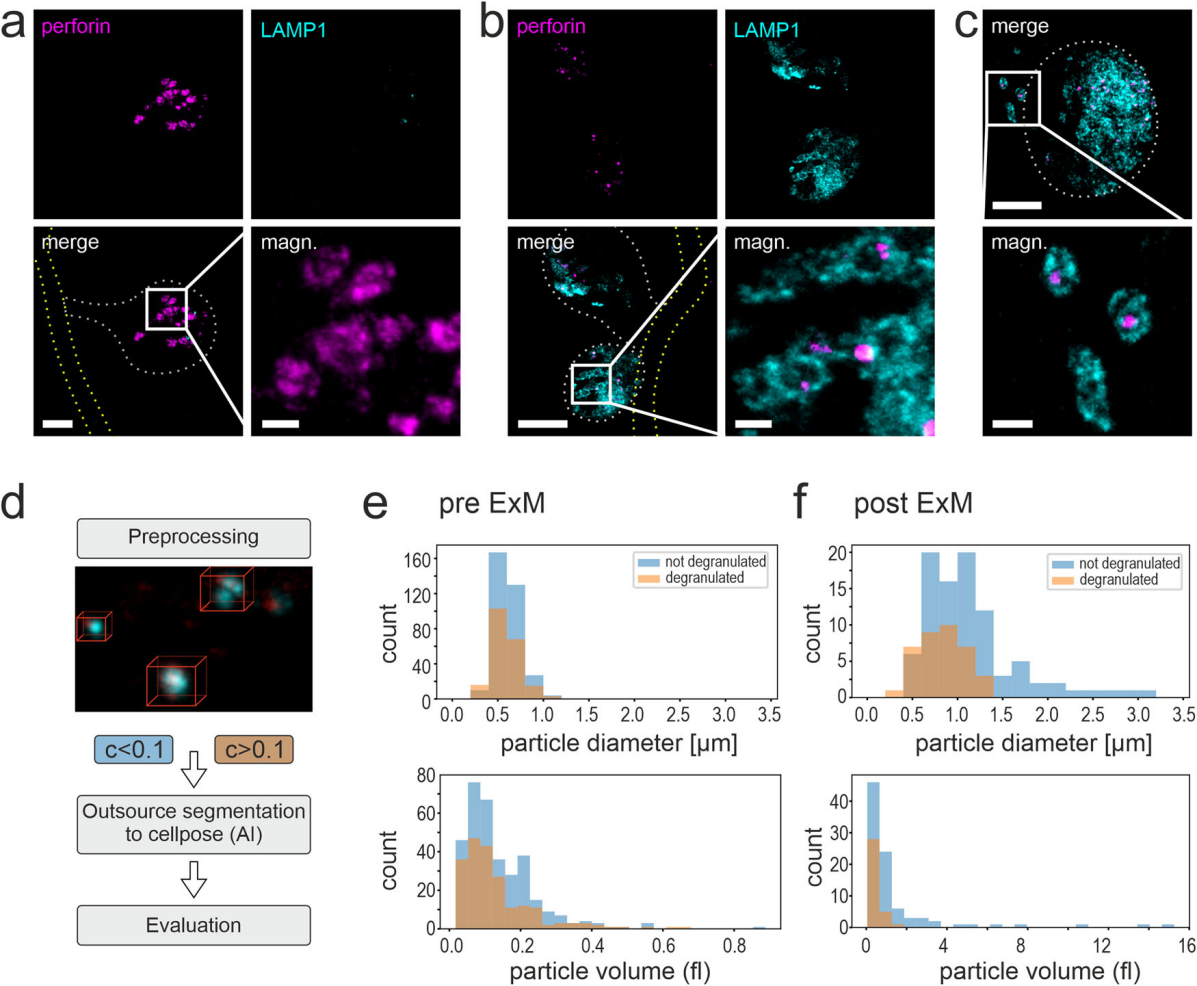
Visualization of NK-cell degranulation by ExM and computation analysis of granule size pre-/post-ExM. Non-degranulated NK cells (**a**) that remain negative for LAMP1 surface marker protein (cyan) show perforin structures (magenta) in a ring-like shape (see a magnification of the indicated box for details). Degranulated NK cells (**b**, **c**) show a high concentration of LAMP1 protein at the surface during degranulation (**b**) and perforin surrounded by LAMP1 is frequently observed (**c**). Note the differences in the size of perforin accumulation in **a** and **b**, **c**. NK cells were cocultured with *A. fumigatus* hyphae for 2.5 h and then treated with BFA for 2 h. All images shown represent maximum intensity z-projections of all slices with exception (magn. in **b**, z projection of slices 9–14). Dotted lines indicate borders of NK cells (gray) and fungal hyphae (yellow). Scale bars, 10 µm (**a**–**c**) and 2 µm in magnifications (magn.). Representative images of two biological replicates (**a**–**c**). **d** Computational analysis workflow. Volumes of interest (VOI) were identified in the perforin channel by a connected component analysis and compared with the LAMP1 signal. Volumes that had more than 10% correlating voxels in the LAMP1 channel were judged as degranulated. VOI were further segmented by cellpose. The volume was computed using a convex hull algorithm. For diameter calculation, a spherical shape of particles was assumed. **e**, **f** Histogram showing the diameter (top) and volume (bottom) distribution of degranulated and non-degranulated particles before (**d**) and after (**e**) expansion. **e** Before the expansion, there is no noticeable difference in volume or diameter. Note that diameters are close to the resolution limit, especially regarding the sampling rate in the z-direction. **f** After expansion, degranulated particles exhibit lower volume and lower diameter than non-degranulated particles. Clusters are drawn apart and can be separated due to higher resolution.

Though Fig. 4 suggests similar localization of perforin and LAMP1 at the NK-cell surface, conventional CLSM images do not enable a closer analysis of the shape of granules and orientation of perforin and LAMP1 (Fig. 4). Therefore, we used ExM to visualize the LAMP1 mediated polarized degranulation (Fig. 5). Similar as with conventional CLSM, also with ExM, the perforin signal was very strong while the LAMP1 signal was almost absent in NK cells before degranulation (Fig. 5a). In contrast, after degranulation, the perforin signal decreased and the LAMP1 signal became prominent (Fig. 5b). Of note, with ExM, we could visualize perforin enclosed by ring-like granules marked by LAMP1 protein at the NK-cell surface. In these measurements, the RFP signal of the already damaged mitochondria was very weak and therefore not shown.

We determined the granule size based on automated image analysis of the perforin signal (Fig. 5c) and compared the size distribution of NK cells before and after degranulation. Cells were defined as degranulated when colocalization of perforin with LAMP1 was obtained in at least 10% of the voxels. Metadata were obtained from the image stacks after image processing including threshold normalization and binary image formation. Based on the detection of connected voxels by cellpose the volume of the granules was determined using a convex hull algorithm^58^ and the diameter was calculated assuming a spherical shape.

In the analysis of unexpanded samples, we found similar size distribution of perforin granules before and after degranulation. The majority of granules exhibited volumes between 0.05 and 0.2 fl in both cell types. Presumably, the volume of vesicles was overestimated in standard CLSM images, as distances between granules can be far below the subdiffraction limit^59^, and thus, perforin-free spaces may be overseen. This problem could partly be compensated by the use of cellpose, but still, some granules were not well separated during the analysis.

In contrast, in ExM image stacks, we could find clear differences in the granule size, with bigger granules before degranulation and smaller ones after degranulation. After ExM granule volumes ranged between 0.01 and 2 fl (mean 0.36 ± 0.3 fl s.d.) in degranulated NK cells and 0.06 and 16 fl (mean 1.4 ± 2.6 fl s.d.) in non-degranulated cells, exhibiting a mean radius of 0.41 ± 0.12 µm (degranulated) and 0.58 ± 0.27 µm (non-degranulated). Raw data are available from Supplementary Data 1. For comparison of the granule volumes in expanded cells versus unexpanded cells, an expansion factor of about 30 (3.1^3^) has to be taken into account. The reduction of the perforin granules after degranulation is significant. We performed a *t*-test with correction for unequal variance. The *p* value for a difference in particle size was 0.00007 whereas with conventional microscopy no significant differences were found (*p* value 0.16). This finding shows the importance of the increased resolution gained by ExM.

## Discussion

ExM fundamentally changes the perspective of subdiffraction fluorescence imaging. Instead of further improving the microscopy setup, the biological sample itself is expanded to increase the spatial resolution. Since its invention ExM proved to be useful for high-resolution imaging of different cell types and applications^25,29,32,33,60^ In the present study, we explored the suitability of ExM for the analysis of the interaction between immune cells and fungal hyphae.

NK cells play an important role in the immune response against filamentous *A. fumigatus*, especially of the hyphal form, as shown for different fungi, including species of *Aspergillus*, *Candida*, and *Lichtheimia*^12^. Therefore, we aimed in evaluating, to what extent the simultaneous expansion of human NK cells and *A. fumigatus* is feasible. Moreover, we investigated how ExM can be advantageously used for super-resolution imaging of immune cell–fungus interactions.

Indeed, after optimization of the ExM protocol for both species and different target structures of interest, we succeeded in simultaneous expansion and visualization of human NK cells interacting with a fluorescent strain of *A. fumigatus*. Overall, we achieved expansion factors of 3.1 to 3.8-fold and successfully visualized different target structures in the NK cell. Our findings demonstrate the potential of the method for the refined study of immune cell–fungi interactions with a multicolor, subdiffraction spatial resolution (Fig. 1). Besides fungal mitochondria, we visualized actin and tubulin in NK cells (Fig. 2) as representatives of the cytoskeleton. As target structures of NK cells’ polarized degranulation we showed perforin together with granulysin (Fig. 3) as well as LAMP1 (Figs. 4, 5). With exception of actin, in all examples, ExM proved to resolve cellular structures in more detail and provide more information about the interaction of the parameters hidden in fluorescence imaging experiments of unexpanded samples.

Microtubules were still resolved when they were about 50 nm apart with ExM while in contrast, the minimal distance was 150–200 nm with standard confocal microscopy. The advantage of ExM for the study of NK cell–fungus interactions can be highlighted by the analysis of granules. Granules shape was clearly resolved revealing colocalization of granulysin and perforin (Fig. 3). Correlation coefficients obtained with pre- and post-ExM are similar and agree within their uncertainties. In contrast, while in standard CLSM images only fluorescent dots can be distinguished, expanded images allowed us to extract valuable information about protein density and shape of the granule.

For the exact determination of distances in the sample, recalculation is required, to obtain the exact size of the measured structure. Similar as in previous ExM experiments that showed isotropic expansion for many different cell types and tissues^34,38^, the elastix analysis indicates, that with our simultaneous protocol the expansion of both, fungi and NK cells, occurs almost isotropically. The correlation between pre-ExM and post-ExM images in linear distortion analysis of NK-cell tubulin was relatively high as reflected by a mean Pearson correlation coefficient of 0.72 ± 0.11 (*n* = 8, Supplementary Fig. 4). Correlation allowing for nonrigid correction only slightly increased the factor to 0.79 ± 0.11, indicating low levels of anisotropy during expansion. In fungi, the correlation of pre- and post-ExM images was a bit lower, but again the correlation by rigid distortion analysis (0.57 ± 0.16; *n* = 4, Supplementary Fig. 3) was similar to nonrigid correction (0.68 ± 0.11).

However, a comparison of the same biological sample before and after expansion revealed that expansion of target structure differed between NK cells (alpha-tubulin, expansion factor 3.06 ± 0.11) and *Aspergillus* hyphae (mito RFP, 3.7 ± 0.11). In addition, we noticed slightly different expansion for different samples or cells in the same sample, indicating that the expansion did not occur completely isotropic (Supplementary Figs. 4, 5). This observation is in accordance with a recent finding, that the expansion factor may depend on the texture of the cell and the nature of the respective organelle^44^. Such differences in expansion factors, whether species-specific or due to a different molecular architecture of the target structure, may provoke artifacts when analyzing the interaction zone of NK cells and *A. fumigatus* hyphae. In our experiments, fungal structures (mitochondria) were not located in the direct environment of the target structures of the NK cells (cytoskeleton, granules). However, visualized labels located at the direct interface should be analyzed with care, as the influence of anisotropy might notably affect the subsequent analysis.

A prerequisite for isotropic expansion is the homogenization by proteinase K that digests the protein content in the sample. As a drawback of this treatment, also fluorescent proteins are affected, though they are relatively stable in the presence of this proteinase^60^. In addition, proteinase K treatment efficacy is known to vary between species and was even used to differentiate bacterial species, due to their varying proteinase K resistance.^61^. Fungal hyphae were typically only treated for 1 h with proteinase K before expansion to preserve fluorescent proteins^38^. Nevertheless, due to the absence of a cell wall and their restrict cytoskeleton^62^, mammalian cells might require longer treatment with proteinase K. Indeed, homogenization for 6 h was required for isotropic expansion of NK cells, but still preserved mRFP signal for visualization of fungal hyphae. Further increase of proteinase K digestion may be enabled using fluorescent tags in the fungus that are more resistant to this critical step before expansion.

Lytic granules released by NK cells are important for the control of fungal invasion. Perforin and granulysin are responsible for the direct NK-cell cytotoxicity towards fungal pathogens, showed for *A. fumigatus* and *R. oryzae* after pretreatment of human NK cells with concanamycin A (ConA) (reviewed in ref. ^11^). However, it has not been fully elucidated to date how perforin or granulysin act together in the killing of fungi by NK cells. Both proteins disrupt the target membranes, leading to a disbalance in ion homeostasis, the influx of water, and loss of intracellular compounds resulting in cell lysis or apoptosis. While granulysin especially attacks membranes lacking cholesterol, perforin is directed against cholesterol-enriched membranes leading to cell lysis or apoptosis. Though fungi enrich ergosterol but not cholesterol in their membranes, purified perforin was shown to damage *A. fumigatus* hyphae and metabolism of *C. albicans*, suggesting the importance of perforin for the antifungal activity of NK cells^63,64^.

In Fig. 3 we noticed a ring-like arrangement of perforin. This is astonishing, as according to the current knowledge one would expect an equal distribution of granzyme and perforin within the granule. Perforin monomers are stored in the lumen of the granule. Upon release in the IS, perforin molecules are exposed to high calcium concentration that triggers a conformational change in the protein structure, and in consequence, allows for incorporation in the target membrane^65^. We may conclude that the concentration of perforin molecules is higher at the surface of the granules due to a so far unknown mechanism. In line with our finding, in recent EM images of perforin-immunostained NK-cell sections, enriched antibody-concentration next to the vesicle membrane can be observed in some of the granules^59^. Nevertheless, we can not exclude that this ring-like appearance is a result of our immune fluorescence staining protocol, a pre-labeling approach that uses primary and secondary antibodies for target structure visualization prior to gelation. Pre-labeling might result at least partly in insufficient staining, as proteins in crowded compartments, like granules, might not be fully accessible for antibodies and thus remain unstained. Another explanation might be that during the initial mild fixation step in RPMI a slight calcium increase occurred in the cell, that could have altered the perforin structure instantly before fixation, potentially leading to partial interaction with the granule membrane of some monomers, that are consequently enriched at the granule membrane leading to the observed phenomenon.

We used BFA that impairs the intracellular trafficking and movement of secretory proteins from the endoplasmic reticulum to the Golgi apparatus^66^. In suchlike treated cells, with ExM we could show that LAMP1 is exposed at the surface of the NK cell after degranulation and that perforin is surrounded by the granule membrane (visualized by LAMP1, an important marker for degranulation^55^), from where it is released to the target cell. In contrast, in pre-ExM images of polarized degranulation, we could only in very exceptional cases guess that perforin might be surrounded by LAMP1, showing the clear advantage of ExM in deciphering fluorescence structures that are close together. Our finding is in accordance with the previous findings, that showed colocalization of the 9-kDa form of granulysin with LAMP1, granzyme B, and perforin in granules of human NK cells^19^. However, the BFA treatment negatively affected the fixation and staining of tubulin as well as the visualization of mito RFP in fungi.

Using ExM we discovered, that the size of the granules is significantly reduced following degranulation. We may explain the absence of bigger granules after degranulation by the fact, that the perforin deposits have been emptied and new granules have to be formed—either by recycling of material or new production—that initially exhibit small volumes. Another possible explanation is that the granules fuse before the attack of the target cell, leaving only smaller, unfused granules in the cell. Nevertheless, exocytosis of lytic granules is size-limited, as the cortical acting layer at the IS hampers provides a mechanical hindrance for granules above 300 nm diameter^59^.

ExM of NK cell/fungus interactions exhibits still limitations that have to be solved in the future. For example, the protocol has to be further improved for the visualization of CD56, an NK-cell pattern recognition receptor for *A. fumigatus*, that directly interacts with the fungal cell wall^16^. Furthermore, the protocol may require adaption when proteins within the fungus serve as the structure of interest and should be labeled with fluorescently labeled antibodies. We noticed that antibodies tend to interact unspecifically with the fungal cell wall quite frequently, as shown in Fig. 2e. In order to enable intrafungal staining with antibodies, the protocol may be adapted to maintain cell wall digestion before the staining procedure, allowing the perfusion of the antibodies to the fungal membrane or into the fungal cytosol.

In conclusion, we demonstrate, that refined ExM is a valuable tool to study NK cell/*A. fumigatus* interactions with high spatial resolution on standard (confocal) fluorescence microscopes. Currently, new variants of ExM including pan-ExM and Sphingolipid-ExM are analyzed in different labs^30,67^ enabling the expansion and visualization of all proteins and sphingolipid-containing cellular membranes with high spatial resolution. In the future, these methods would be of advantage to further improve our knowledge of IS formation between NK cells and *A. fumigatus* hyphae, a complex process, involving several proteins as well as dynamic changes in lipid composition of the NK-cell membrane

## Methods

### Fungal culture

An *A. fumigatus* strain expressing mitochondria-targeted red fluorescent protein (RFP) under control of the *Aspergillus nidulans gpdA* promoter was kindly provided by J. Wagener^38^. *A. fumigatus* spores were harvested as described before^38^ and plated on KOH-treated glass coverslips (Carl Roth, # YX03.1, 12 mm diameter) in a 4x-well plate format (Nunc, # 176740) for overnight incubation at 30 °C in RPMI medium (Sigma, # R7509-500ML). Small germlings were used for coculture experiments.

### NK-cell preparation

Peripheral blood mononuclear cells (PBMCs) were isolated from healthy blood donors using leukoreduction system (LRS) chambers, obtained from the University Hospital Wuerzburg (UKW) from the Institute of Transfusion Medicine and Haemotherapy. Usage of the human blood specimens was approved by the Ethical Committee of the University Hospital Wuerzburg.

PBMC´s were isolated using Histopaque®−1077 density gradient centrifugation. In detail LRS blood (~10 ml) was mixed with HBSS (Sigma, # H6648-500ml), supplemented with 1% FCS (Sigma, F7524-500ml) and 2 mM EDTA (Sigma, # E7889-100ml), to a final volume of 50 ml. A volume of 25 ml blood mixture was carefully layered onto 20 ml Histopaque®-1077 solution (1.077 g/ml density, Sigma, Histopaque®-1077, # 10771-500 ml). Centrifugation was performed for 20 min at 800x*g* with the lowest acceleration/deceleration settings at room temperature (RT). Buffy coat was harvested using a Pasteur-pipette and transferred in a 50 ml tube. Prior to centrifugation (120x*g*, 15 min, lowest acceleration/deceleration settings), the tube was topped up with HBSS to a final volume of 50 ml. This step was performed twice before counting PBMC´s via a Cell Viability Counter (Vi-cell ™ XR, Beckman Coulter).

PBMC´s were adjusted to 1 × 10^8^ cells/400 µl for NK-cell negative-selection using NK-Cell Isolation Kit human (Miltenyi Biotec, # 130-092-657). The isolation was performed according to the manufacturer’s protocol.

For NK-cell cryo conservation, cells were centrifuged (300x*g*, 10 min at RT, highest acceleration/deceleration settings) and resuspended in ice-cold freeze-mix (90% FCS + 10% DMSO) to a final concentration of 3 × 10^6^ cells/ml (DMSO, Roth, # A994.2 (250 ml)). Cryotubes were transferred into a Freezing Container (Mr. Frosty) and cooled down to −80 °C for 24 h. Frozen cryotubes were stored in liquid nitrogen until use.

### NK-cell culture

NK cells were thawed using a preheated water bath (37 °C) and diluted in 10 ml RPMI-1640 (Sigma, # R8758-500ML) medium, supplemented with 10% FCS. For washing, NK cells were centrifuged at 300x*g*, 10 min at RT with the highest acceleration/deceleration settings. Cells were adjusted to 1 × 10^6^ cells/ml and transferred into a 6-well plate format. For overnight incubation 20 µl ProL/ml (1000 U/ml ProL, Proleukin-S, Novartis) were added and cells were incubated at 37 °C and 5% CO_2_. For coculture experiments, NK cells were centrifuged the next day (same settings) and resuspended in RPMI-1640 w/o ProL.

### NK cell/*A. fumigatus* coculture

NK cells were cocultured with *A. fumigatus* germlings in RPMI-1640 (10% FCS) at 37 °C and 5% CO_2_ on 12-mm-glass coverslips. *A. fumigatus*/NK cell ratio was adjusted to a multiplicity of infection (MOI) of 0.4–0.5. Co-incubation time was set to 5–5.5 h in all experiments shown if not stated otherwise.

### NK-cell immobilization on poly-d-lysine coated glass coverslips

For NK-cell preparation w/o *A*. *fumigatus* hyphae, 12-mm-glass coverslips were coated with 0.05% PDL (Sigma, # P6407-5MG). NK cells were settled on top (200,000–250,000 cells per well, four well plate). For recovery NK cells were incubated for another hour at 37 °C and 5% CO_2_ prior to immune fluorescence staining.

### Fixation and staining procedures

For fixing and permeabilizing the cells formaldehyde (FA, Sigma, Cat # F8775), GA (Sigma, Cat # 354400, 25% aqueous sol), and saponin (Quilljabark, Sigma, Cat # S-7900, Lot: 91H0325) were used.

### Staining of cytoskeleton structures

#### Alpha-tubulin

After NK cell/*A. fumigatus* co-incubation the medium was replaced by a permeabilization buffer containing 0.25% GA and 0.25% Triton-X-100 in 10 mM MES pH 6.1, 150 mM NaCl, 5 mM EGTA, 5 mM glucose, and 5 mM MgCl_2_ (37 °C pre-warmed, 1 min). Followed by fixation with 2% GA using the same buffer w/o Triton-X-100 (RT, 10 min). After washing twice in 1x PBS the sample was blocked in 5% BSA/PBS for 30 min at RT. Primary rabbit anti-alpha-tubulin (Abcam, # ab18251) was diluted to 5 µg/ml in 5% BSA/PBS and incubated for 1 h at RT. After washing twice in 0.1% Tween/PBS, secondary goat anti-rabbit Alexa Fluor 488 ab (Invitrogen, # A11070, fab fragment) was diluted to 10 µg/ml in 5% BSA/PBS and incubated for 1 h at RT. After washing twice with Tween/PBS, the sample was postfixed in 2% FA/0,25%GA in PBS, 10 min at RT.

#### Actin

After NK cell/*A. fumigatus* co-incubation the medium was replaced by a permeabilization buffer containing 0.25% GA and 0.25% Triton-X-100 in 10 mM MES pH 6.1, 150 mM NaCl, 5 mM EGTA, 5 mM glucose, and 5 mM MgCl_2_ (37 °C pre-warmed, 1 min). Followed by fixation with 2% GA using the same buffer w/o Triton-X-100 (RT, 10 min). After washing with 1x PBS the sample was blocked in 2.5% BSA/PBS for 30 min at RT. Phalloidin-XX-biotin (PromoKine, # PK-CA707-00028) was diluted in blocking solution to 0.5 µM and incubated for 1 h 20 min at RT. The sample was washed twice with blocking solution, followed by incubation with Streptavidin ATTO 643 (ATTO TEC, # AD643-61) for 1 h at RT (10 µg/sample). After washing twice in 1x PBS, the sample was postfixed in 0.25% GA/PBS for 15 min at RT.

### Staining of perforin and granulysin

NK cell/*A. fumigatus* cocultures were fixed by topping up medium with a final concentration of 0.7% FA/RPMI (10% FCS) post 5.5 h cocultivation. After washing with 1x PBS, the sample was blocked in 2.5% BSA in 0.1% saponin/PBS, 30 min, RT. Primary rabbit anti granulysin ab (Abbexa, # abx006369) and mouse anti perforin ab (BioLegend, # 308102) were diluted to 10–25 µg/ml, respectively and incubated for 1.5 h at RT. After two washing steps in 2.5% BSA in 0.1% saponin/PBS, secondary goat anti-rabbit Alexa Fluor 488 (Invitrogen, # A11070) and goat anti-mouse ATTO 643 (sigma, # SAB3701063, custom labeled with ATTO 643) were diluted to 10 and 5.5 µg/ml, respectively and incubated for 1 h at RT. After washing twice in 2.5% BSA in 0.1% saponin/PBS the sample was postfixed using 3.7% FA/0.25% GA/PBS, 10 min, RT.

### NK-cell degranulation assay—staining of surface LAMP1 and perforin

After 3 h of NK cell/*A. fumigatus* co-incubation the medium was topped up by RPMI (10% FCS) containing BFA, 5 µg/ml final concentration (BioLegend, # 420601) and primary rabbit anti LAMP1 ab, 8.3 µg/ml final concentration (Abcam, # ab24170). Coculture was continued for another 2 h. Sample fixation was performed by adding FA/RPMI (10% FCS) for 5 min at 37 °C with a final concentration of 1.2% FA. A second fixation step in 3% FA/RPMI (10% FCS) for 5 min at 37 °C was performed, followed by three washing steps in 0.1% Saponin/PBS. Sample blocking was performed in 5% BSA/PBS for 30 min at RT. Primary mouse anti perforin ab (BioLegend, clone dG9, # 308102) was diluted to 10 µg/ml in 0.1% Saponin/PBS and incubated for 1 h at RT, followed by three washing steps in 0.1% Saponin/PBS. Secondary goat anti-mouse Alexa Fluor 488 ab (Thermo Fisher, # A11017, fab fragment) was diluted to 10 µg/ml in 0.1% Saponin/PBS and incubated for 1 h at RT. After washing twice in 0.1% Saponin/PBS secondary donkey anti-rabbit ATTO 643 (Jackson ImmunoResearch, # 711-005-152, custom labeled with ATTO 643) was diluted to 5 µg/ml in 5% BSA/PBS for 1 h at RT. After washing twice, the sample was postfixed with 3.7% FA/0.25% GA in PBS, 10 min at RT.

### Cell wall lysis, proteinase K digestion, and expansion

Cell wall lysis stock solution, containing 0.2 g Lysing Enzyme (Sigma, # L1412-10G, *Trichoderma harzianum*) and 1 mg Chitinase (sigma, # C6137-5UN, *Streptomyces griseus*) per 20 ml 0.7 M NaCl (Sigma, # S5886), was diluted 1:2 in 0.7 M NaCl for *A. fumigatus* cell wall digestion. Cell wall lysis stock solution was stored at −80 °C in 400-µl-aliquots until use. *A. fumigatus* cell wall lysis was performed for 1 h at RT after IF and postfixation, prior to sample gelation.

After cell wall lysis the sample was rinsed in 1x PBS followed by gelation. For sample gelation, 85 µl monomeric solution [8.625% sodium acrylate (Sigma, # 408220), 2.5% acrylamide (Sigma, # A9926), 0.15% *N*,*N*′-methylenbisacrylamide (Sigma, # A9926), 2 M NaCl (Sigma, # S5886), 1x PBS, and 0.2% freshly added ammonium persulfate (APS, Sigma, # A3678) and tetramethylethylenediamine (TEMED, Sigma, # T7024)] was placed as droplet on top of a parafilm-stripe. The coverslip was placed on top of the droplet, facing upside down. Gelation took place in a humid chamber overnight, in darkness at RT. For sample orientation, the gel was cut to a SIM-card format and placed in digestion buffer [50 mM Tris pH 8.0, 1 mM EDTA (Sigma, # ED2P), 0.5% Triton-X-100 (Thermo Fisher, # 28314), and 0.8 M guanidine HCl (Sigma, # 50933)] supplied with 8 U/ml proteinase K (Thermo Fisher, # AM2548). Proteinase K digestion was performed for 6 h at RT, followed by expansion in ddH_2_O overnight at 8 °C. All steps were performed in darkness. Expanded gels were stored in ddH_2_O at 8 °C in darkness.

### Confocal laser scanning microscopy

Confocal scanning microscopy was performed using a Zeiss LSM700, equipped with a water immersion objective (C-Apochromat 63x/1.20 W Korr M27).

For accurate z sectioning, fully expanded sodium acrylate gels were immobilized using poly-d-lysine (Sigma, # P6407-5MG) coated 1x-well chambers (Nunc, # 155360, #1.5 borosilicate). To prevent gel drying, a humid tissue was placed within the chamber during imaging. For excitation of ATTO 643 conjugates, the 639 nm laser line was used: pre-expansion: 1,5–4% laser int., 600–650 digital gain and post expansion: 6–7% laser int., 650 digital gain. For phalloidin-XX-biotin/Streptavidin ATTO 643 conjugates, the 639 nm laser line was used with 30% int. post expansion. For excitation of Alexa Fluor 488 conjugates, the 488 nm laser line was used: pre-expansion: 1–3% laser int., 550–600 digital gain, and post expansion: 5–7% laser int., 600 digital gain. For excitation of the RFP conjugate (*A. fumigatus* mito RFP) the 555 nm laser line was used: pre-expansion: 3–4% laser int., 600 digital gain and post expansion: 5–6% laser int., 650–700 digital gain. Sectioning was achieved using ZEN software. The creation of maximum intensity z-projections was performed using Image J (FIJI, Wayne Rasband, NIH^68^).

### Automated volume computation for expanded and unexpanded cells

LAMP1 (cyan) and perforin (magenta) color channels of Image *I* were used for evaluation. *I* was processed with a gamma correction (γ = 0.9) and normalized using equation 1: ${I}_{{new}}=(I/{\max }(I))-{th}$. To reduce the computational cost of artificial intelligence (AI) based segmentation, we identified and cropped volumes of interest by applying a connected components analysis^69^, implemented in scikit- image^70^. For further segmentation, we implemented cellpose^71^, with the pretrained “nucle” model. Cellpose requires an anisotropy parameter describing the relation of planar to axial resolution, which was computed with equation 2: $a=\frac{{{{{{{{\mathrm{pixel}}}}}}\_{{{{{\mathrm{size}}}}}}}}_{x}}{{{{{{{{\mathrm{pixel}}}}}}\_{{{{{\mathrm{size}}}}}}}}_{z}}$. The estimated cell diameter parameter was set to the diameter of the initial segmentation. The process yielded a label image *L*, where the voxels of estimated cells are marked with corresponding integer number ${m}_{i}$ and can thus be separated. We computed the cell volume using a convex hull algorithm. The two populations were separated using the correlation of perforin and LAMP1. Particles exceeding a correlation value of 10% were regarded as degranulated.

### Distortion analysis and determination of expansion factors

To evaluate the quality of our expanded samples we implemented a workflow with three steps: First, we used Elastix^42^ to compute a similarity transform that maps the pre-expansion image to the post expansion image. i.e., we maximized the overlap of a signal under a transformation with the given degrees of freedom (DOF). A similarity transform includes four DOF, namely a rotation, the translations in x and y direction, and scalation. Therefore, the mapping only compensates for the position under the microscopy and isotropic expansion in all directions. To validate the transform, we compute a Pearson correlation index, a measurement for similarities in two images^72^. In a second step, we computed a B-spline transform of the transformed pre-expansion image to estimate the degree of nonlinearities in the expansion. A B-spline transform is very flexible, having two DOF for every pixel in the image, and is, therefore, suitable to compensate nonlinear distortions. Applying this transform results in a better alignment and the difference in Pearson correlation indices already indicates the linearity of the expansion. In a third step, the computed B-spline transform can be used to compute a distortion map. The red arrows in Supplementary Fig. 2d represent the vectorial shift necessary to correct the remaining distortions after the application of the similarity transform. We wrote a custom script implementing the workflow in python^73^.

### Statistics and reproducibility

Information on the research design and reproducibility is available via the Nature Research Reporting Summary linked to this article, including sample sizes, number of replicates, and replicate definition.

### Reporting Summary

Further information on research design is available in the Nature Research Reporting Summary linked to this article.

## Supplementary information

Supplementary Information

Description of Additional Supplementary Files

Supplementary Data 1

Reporting Summary

## Data Availability

All relevant data are included in the manuscript and the supplementary information. Image stacks used in Figs. 2–5 and supplementary information can be downloaded from Figshare. 10.6084/m9.figshare.c.5577690.v1^73^. Further image stacks are available from the corresponding author upon reasonable request.

## Code availability

Source code for Perforin/Lamp1 evaluation is available at https://github.com/super-resolution/PerforinLamp1Evaluation. All packages that were used and the corresponding versions are declared in the requirements.txt file. Custom script implementing the distortion analysis workflow in Python is available from Zenodo: https://zenodo.org/record/5234670.^73^
