# Supplementary material for: Subdiffraction-resolution fluorescence imaging of immunological synapse formation between NK cells and A. fumigatus by expansion microscopy
Source: Commun Biol. 2021 Oct 4;4:1151. doi: 10.1038/s42003-021-02669-y (PMC8490467; doi:10.1038/s42003-021-02669-y)
Supplement: Supplementary file 1 — Supplementary Information [file 42003_2021_2669_MOESM1_ESM.pdf]

Supplementary Information

Subdiffraction-resolution fluorescence imaging of immunological synapse formation between NK cells and *A. fumigatus* by expansion microscopy

Nora Trinks[1], Sebastian Reinhard[1], Matthias Drobny[2], Linda Heilig[2], Jürgen Löffler[2], Markus Sauer[1], Ulrich Terpitz[1,*]

[1] Department of Biotechnology and Biophysics, Theodor-Boveri-Institute, Biocenter, Julius Maximilian University, Würzburg, Germany

[2] Department of Internal Medicine II, WÜ4i, University Hospital Würzburg, Würzburg, Germany

* e-mail: ulrich.terpitz@uni-wuerzburg.de

a

	pre expansion [mm]	post expansion [mm]	estimated expansion factor
A → B	6	23	~ 3,83
C → D	9	35	~ 3,89
diameter	12	49	~ 4,08
mean ± SD			~ 3,94 ± 0,13

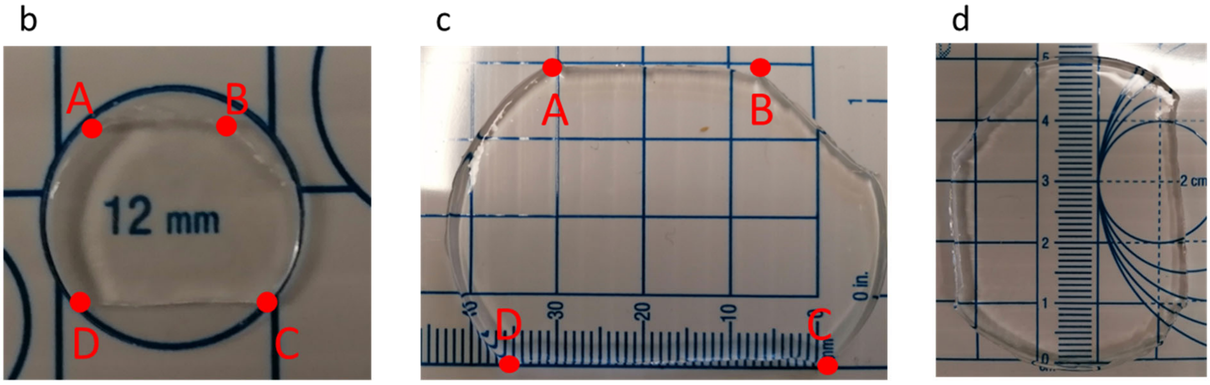

Supplementary Figure 1. Macroscopic expansion factor determination.

Table (a) shows measured gel lengths of the same gel cut to a SIM-card format (gel pre expansion shown in b and gel post expansion shown in c and d). A diameter of 12 mm was determined after gelation over night (b). A diameter of 49 mm was determined post expansion in ddH_2O (d).

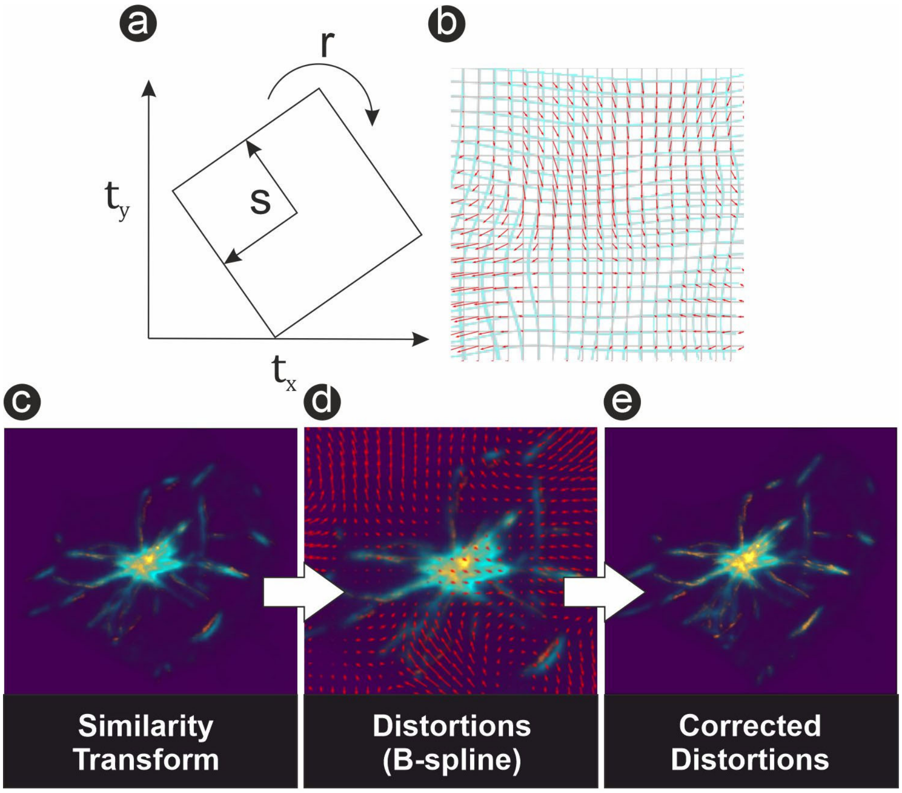

Supplementary Figure 2. Workflow for the determination of structural Expansion

In a first step the pre-expansion image (cyan) is rescaled with the expected expansion factor and registered over a similarity transform (**c**) with the post-expansion image (orange). A similarity transform has only 4 degrees of freedom (**a**), t_x and t_y the translation in x and y direction. A rotation r and a scaling s. Therefore, it only corrects distortions present in isotropic expansion. The registered image is used to compute nonlinear distortions with a B-spline transform (**b**). The red arrows indicate the local offset from the orange to the cyan channel (**d**). Applying these distortions results in an improved overlay (**e**).

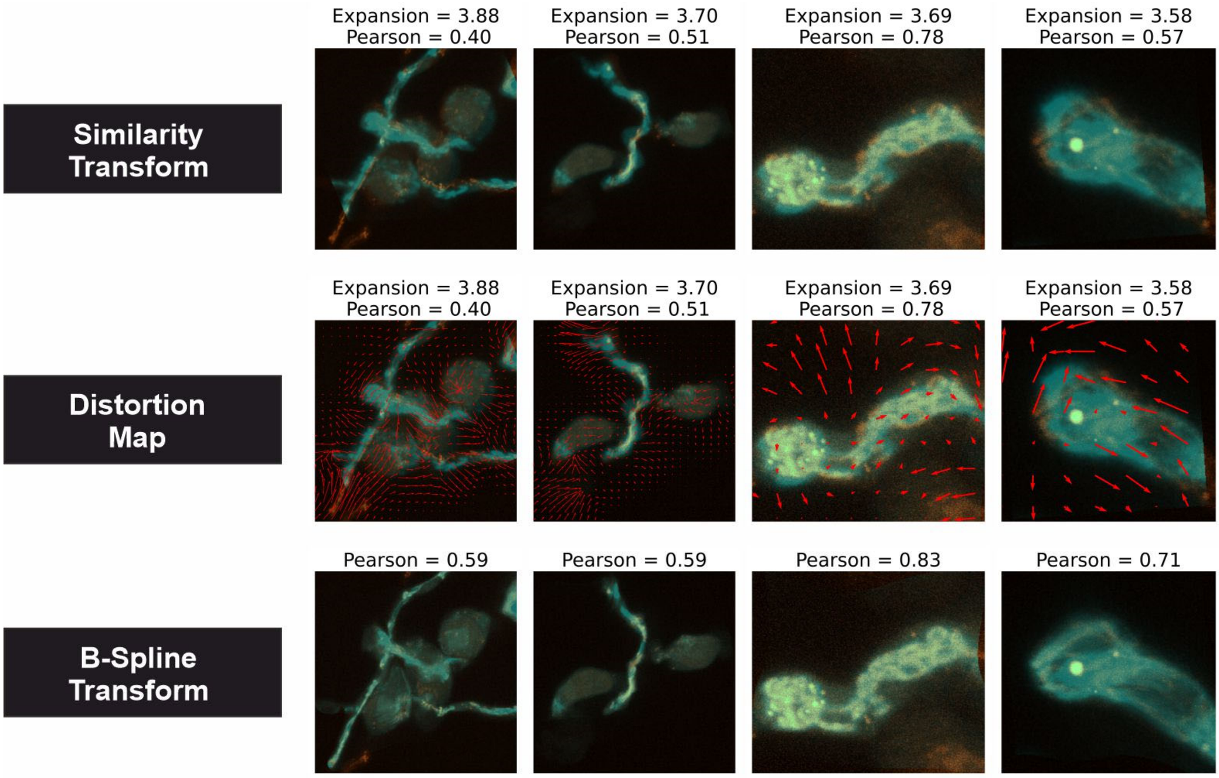

Supplementary Figure 3. Determination of expansion factor for mitoRFP in *A. fumigatus.* The similarity transform aligns the images as well as possible for the 4 given DOFs. The Distortion map shows further nonlinear adjustments to improve the overlay. Red arrows indicate the vectorial shift applied to a position to align the images as shown in the b-Spline transform series.

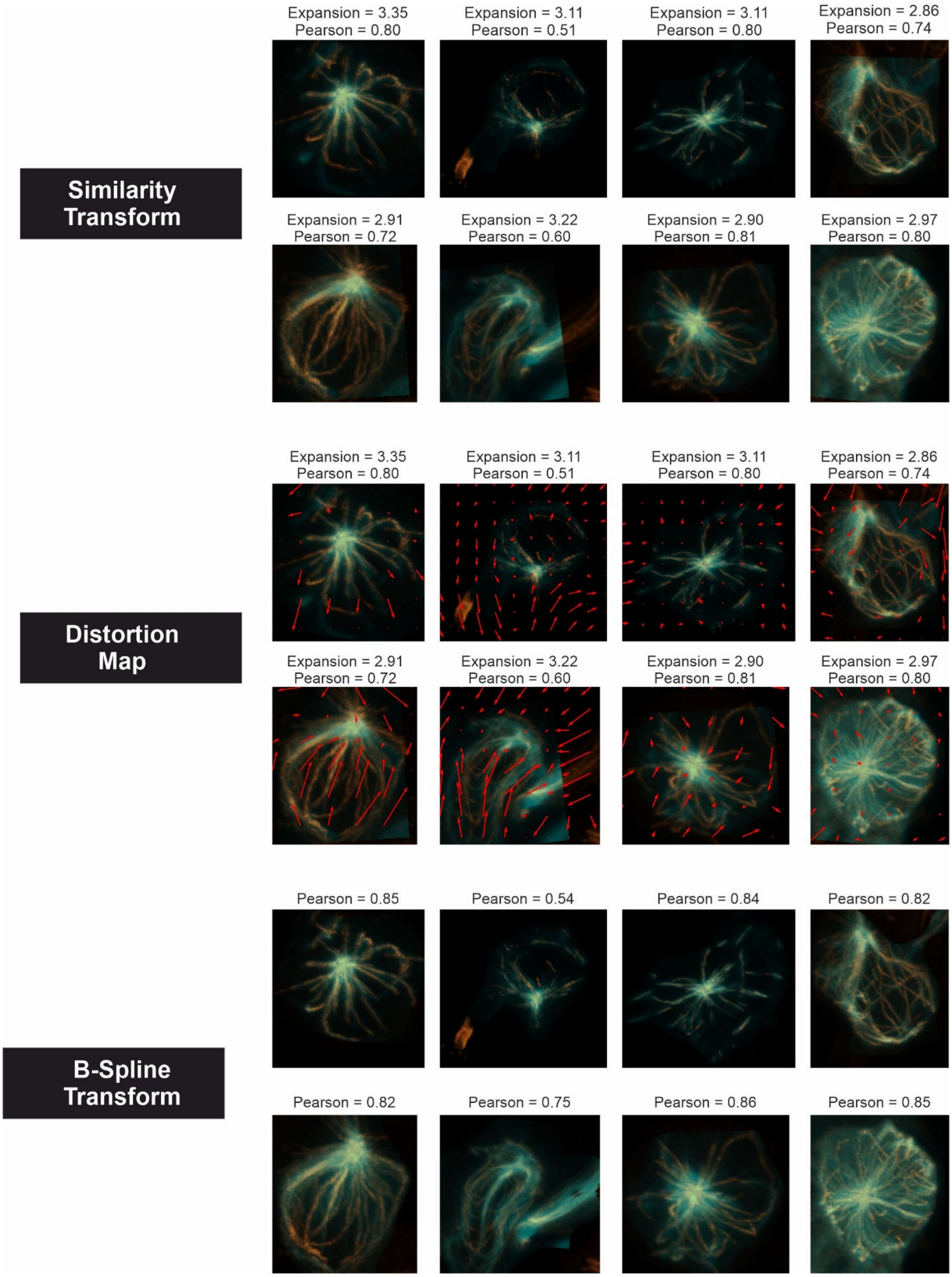

Supplementary Figure 4. Determination of expansion factor for alpha-tubulin in NK cells. The similarity transform aligns the images as well as possible for the 4 given DOFs. The Distortion map shows further nonlinear adjustments to improve the overlay. Red arrows indicate the vectorial shift applied to a position to align the images as shown in the b-Spline transform series.

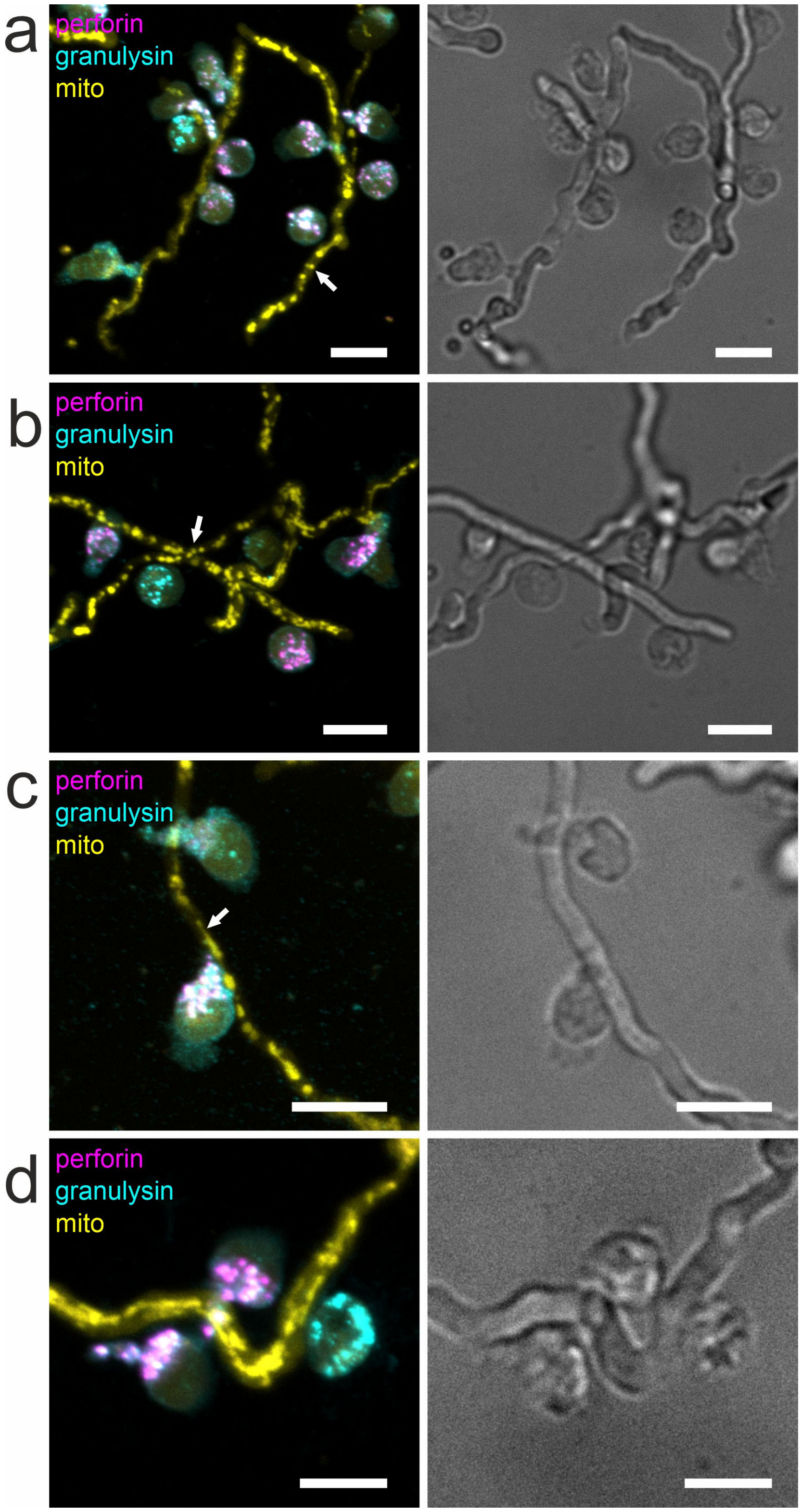

Supplementary Figure 5. Degradation of Mitochondria next to degranulating NK cells.

CLSM images (left) and corresponding bright field images (right) of NK cells interacting with *A. fumigatus* mito-mRFP (yellow) stained for granulysin (cyan) and perforin (magenta). In the direct environment of degranulated NK cells, often (**a-c**) but not always (**d**) the mitochondrial morphology is impaired. All images represent maximum intensity z-projection images, Scale bars 10 µm.
