# Supplementary material for: Subdiffraction-resolution fluorescence imaging of immunological synapse formation between NK cells and A. fumigatus by expansion microscopy
Source: Commun Biol. 2021 Oct 4;4:1151. doi: 10.1038/s42003-021-02669-y (PMC8490467; doi:10.1038/s42003-021-02669-y)
Supplement: Supplementary file 2 — Description of Additional Supplementary Files [file 42003_2021_2669_MOESM2_ESM.pdf]

Description of Additional Supplementary Files

File name: Supplementary Data 1

Description: Raw data of particle diameter.
