# Supplementary material for: Subdiffraction-resolution fluorescence imaging of immunological synapse formation between NK cells and A. fumigatus by expansion microscopy
Source: Commun Biol. 2021 Oct 4;4:1151. doi: 10.1038/s42003-021-02669-y (PMC8490467; doi:10.1038/s42003-021-02669-y)
Supplement: Supplementary file 4 — Reporting Summary [file 42003_2021_2669_MOESM4_ESM.pdf]

nature research

Corresponding author(s): Ulrich Terpitz

Last updated by author(s): Aug 30, 2021

Reporting Summary

Nature Research wishes to improve the reproducibility of the work that we publish. This form provides structure for consistency and transparency in reporting. For further information on Nature Research policies, see our Editorial Policies and the Editorial Policy Checklist.

Statistics

For all statistical analyses, confirm that the following items are present in the figure legend, table legend, main text, or Methods section.

n/a	Confirmed	
☐	☒	The exact sample size (*n*) for each experimental group/condition, given as a discrete number and unit of measurement
☐	☒	A statement on whether measurements were taken from distinct samples or whether the same sample was measured repeatedly
☐	☒	The statistical test(s) used AND whether they are one- or two-sided<br>*Only common tests should be described solely by name; describe more complex techniques in the Methods section.*
☒	☐	A description of all covariates tested
☒	☐	A description of any assumptions or corrections, such as tests of normality and adjustment for multiple comparisons
☒	☐	A full description of the statistical parameters including central tendency (e.g. means) or other basic estimates (e.g. regression coefficient) AND variation (e.g. standard deviation) or associated estimates of uncertainty (e.g. confidence intervals)
☒	☐	For null hypothesis testing, the test statistic (e.g. *F*, *t*, *r*) with confidence intervals, effect sizes, degrees of freedom and *P* value noted<br>*Give P values as exact values whenever suitable.*
☒	☐	For Bayesian analysis, information on the choice of priors and Markov chain Monte Carlo settings
☒	☐	For hierarchical and complex designs, identification of the appropriate level for tests and full reporting of outcomes
☒	☐	Estimates of effect sizes (e.g. Cohen's *d*, Pearson's *r*), indicating how they were calculated

Our web collection on statistics for biologists contains articles on many of the points above.

Software and code

Policy information about availability of computer code

Data collection	For maximum intesity z-projection creation Image J was used (version: ImageJ 1.52i). For image acquisition ZEN 2012 software was used (ZEN 2012 SP1 (black edition, 64 bit), Release Version 8.1).
Data analysis	Source code for Perforin Lamp1 evaluation is available at: https://github.com/super-resolution/PerforinLamp1Evaluation or https://zenodo.org/record/5234670 (DOI:10.5281/zenodo.5234670). All packages that were used and the corresponding versions are declared in the requirements.txt file.

For manuscripts utilizing custom algorithms or software that are central to the research but not yet described in published literature, software must be made available to editors and reviewers. We strongly encourage code deposition in a community repository (e.g. GitHub). See the Nature Research guidelines for submitting code & software for further information.

Data

Policy information about availability of data

All manuscripts must include a data availability statement. This statement should provide the following information, where applicable:

- Accession codes, unique identifiers, or web links for publicly available datasets
- A list of figures that have associated raw data
- A description of any restrictions on data availability

All relevant data are included in the manuscript and the supplementary information. Image stacks used in Figures 2-5 and supplementary information can be downloaded from https://doi.org/10.6084/m9.figshare.c.5577690. Further image stacks are available from the corresponding author upon reasonable request.

Field-specific reporting

Please select the one below that is the best fit for your research. If you are not sure, read the appropriate sections before making your selection.

☒ Life sciences ☐ Behavioural & social sciences ☐ Ecological, evolutionary & environmental sciences

For a reference copy of the document with all sections, see nature.com/documents/nr-reporting-summary-flat.pdf

Life sciences study design

All studies must disclose on these points even when the disclosure is negative.

Sample size

Fig. 2: a. 1 representative image out of 8 stacks (~ 29 cells imaged, n=1). b, c. 2 representative images out of 9 stacks (~ 11 cells imaged, n=1). d. 1 representative image out of 21 stacks (~ 25 cells imaged, n=1). e. 1 representative image out of 20 stacks (~ 23 cells imaged, n=1). f, g. 1 image out of 9 stacks (~ 9 cells imaged, n=1). h. 1 representative image out of 8 stacks (~ 34 cells imaged, n=1). i. 1 representative image out of 9 stacks (~ 9 cells imaged, n=1). k, l, m. 2 representative image out of 12 stacks (~ 9 cells imaged, n=1).

Fig. 3: a. 1 representative image out of 5 stacks (~ 28 cells imaged, n=1). b, c. 2 representative images out of 2 individual experiments (15 stacks with ~ 20 imaged cells and 11 stacks with ~ 11 imaged cells, n=2).

Fig. 4: b, c. 2 representative images out of 10 stacks (~ 50 cells imaged, n=1).

Fig. 5: a, b. 2 representative images out of 11 stacks (~ 13 cells imaged, n=1).

d, e. all data sets that have been stated for Fig 4 b & c and Fig 5 a & b were analysed for volume calculation of degranulated NK-cells and non-degranulated NK-cells, which represents Fig 5 d & e.

Data exclusions

data exclusion when drift was recognised (e.g. single stacks were not taken into observation upon drift recognition).

Replication

For alpha-tubulin staining 5 replicates were done. For actin staining 2 replicates were done. For perforin and granulysin co-staining 3 replicates were done. For LAMP-1/perforin degranulation assay 2 replicates were done.

Randomization

n/a

Blinding

n/a

Reporting for specific materials, systems and methods

We require information from authors about some types of materials, experimental systems and methods used in many studies. Here, indicate whether each material, system or method listed is relevant to your study. If you are not sure if a list item applies to your research, read the appropriate section before selecting a response.

Materials & experimental systems

n/a	Involved in the study	
☐	☒	Antibodies
☒	☐	Eukaryotic cell lines
☒	☐	Palaeontology and archaeology
☒	☐	Animals and other organisms
☐	☒	Human research participants
☒	☐	Clinical data
☒	☐	Dual use research of concern

Methods

n/a	Involved in the study	
☒	☐	ChIP-seq
☒	☐	Flow cytometry
☒	☐	MRI-based neuroimaging

Antibodies

Antibodies used

Primary antibodies:
- Purified anti-human Perforin Antibody, BioLegend, Cat. 308102, clone: dG9, Lot. B267038.
- Granulysin (GNLY) Antibody, abbexa, Cat. abx006369, rabbit polyclonal, Lot. LA2006654Z.
- Anti-alpha Tubulin antibody, abcam, Cat. ab18251, rabbit polyclonal, Lot. GR3293836-1.
- Anti-LAMP1 antibody, abcam, Cat. ab24170, rabbit polyclonal.

Secondary antibodies:
- F(ab')2-Goat anti-Rabbit IgG (H+L) Cross-Adsorbed Secondary Antibody, Alexa Fluor 488, Invitrogen, Cat. A11070, polyclonal, Lot. 2018208.
- Anti-Mouse IgG (H+L), highly cross adsorbed antibody produced in goat, custom labelled with ATTO 643-NH-S, sigma, Cat. SAB3701063, polyclonal.

- AffiniPure Donkey Anti-Rabbit IgG (H+L), custom labelled with ATTO 643-NH-S, Jackson ImmunoResearch, Cat. 711-005-152, rabbit polyclonal, Lot. 139003.
- AffiniPure Donkey Anti-Mouse IgG (H+L), custom labelled with ATTO 643-NH-S, Jackson ImmunoResearch, Cat. 715-005-150, mouse polyclonal, Lot. 140284.

Antibody staining concentrations in [µg/mL] can be found for each antibody type in the appropriate material and methods section.

Validation

All antibodies are commercially available and validation reports can be found on the manufacturers websites, using antibody name and Cat. number (summarized above). Custom labelled secondary antibodies are stated above, validation occured via multiple independent immunofluorescence stainings with appropriate samples.

Human research participants

Policy information about studies involving human research participants

Population characteristics

Human healthy volunteer blood donors, random distribution of sex/gender, >18 years, <65 years, no HIV, hepatitis B, hepatitis C infection detectable

Recruitment

Random recruitment of blood samples, random distribution of sex/gender and age of blood donors. All blood samples were anonymized immediately after taking and prior to processing by laboratory personel.

Ethics oversight

The studies involving human participants were reviewed and approved by ethics committee of the University of Wuerzburg (#34/15). The participants provided their written informed consent to participate in this study.

Note that full information on the approval of the study protocol must also be provided in the manuscript.
